# Why have four decades of research into decreased ovarian reserve (DOR) failed to improve live birth rates?

**DOI:** 10.1007/s00404-026-08550-9

**Published:** 2026-08-05

**Authors:** Zion Ben Rafael, Raoul Orvieto

**Affiliations:** 1 Department of Obstetrics and Gynecology and IVF, Laniado Medical Center, Netanya, Israel; 2 Chaim Sheba Medical Center, Ramat- Gan. The Tarnesby-Tarnowski Chair for Family Planning and Fertility Regulation,the Gray Faculty of Medical and Health Science, Tel-Aviv University, Tel-Aviv, Israel

**Keywords:** IVF, Decreased ovarian reserve, Poor ovarian response, Age, Euploidy, Live birth rate

## Abstract

Despite forty years of intense research and multitudinous clinical innovations, live birth rates (LBR) among women diagnosed with decreased ovarian reserve (DOR) remain disappointingly poor. Is this because a solution has not yet been discovered, or are we searching in the wrong way? This persistent failure is probably the product of both undisputable biological realities, the exponential decline in oocyte euploidy with advanced maternal age, and persistent methodological flaws, most notably the inclusion of highly heterogeneous cohorts in clinical trials and inadequate stratification by critical variables, such as age. No ovarian stimulation protocol, adjuvant therapy, or laboratory add-on has consistently improved outcomes. The grouping of young and older DOR women in clinical studies, the focus on surrogate endpoints over live birth, and failure to account for cycle-to-cycle and operator variability further obscure valid conclusions and mislead both clinicians and patients. In this review, we delineate the evolution of DOR research, drilling down to the biological, technical, and methodological roots of failed progress. We argue that only by rigorously stratifying study populations by narrow age bands, repeated response criteria and prioritizing true endpoints, can future research genuinely advance the field and provide realistic, evidence based counseling. Clinical humility and research rigor are prerequisites for sparing patients needless interventions and false hope.

## Introduction

The persistent inability to substantially improve live birth rates (LBR) in women with decreased ovarian reserve (DOR) remains one of the greatest unresolved challenges in reproductive medicine. Over the past four decades, an explosion of research, innovation, and clinical effort has failed to yield meaningful clinical progress for this group, despite remarkable advances in other areas of assisted reproductive technology (ART). The paradox is striking: while younger women and those with favorable prognostic features now enjoy excellent cumulative outcomes from in vitro fertilization (IVF), women with DOR, especially those of advanced maternal age, continue to face grim odds regardless of protocol, add-ons, or laboratory sophistication [1, 2].

This review undertakes a comprehensive, critical analysis of why four decades of research into DOR have failed to translate into improved LBR. We interrogate not only the well-described biological realities of ovarian aging, the loss of both follicular quantity and oocyte quality, but also the methodological and conceptual flaws that continue to restrict research and mislead both clinicians and patients.

## Background

According to a national cohort study from Denmark [3], the total 5-year live birth rates were strongly influenced by female age and ranged from 80% in women under 35 to 26% in women ≥ 40 years. Yet, the cumulative live birth rate after IVF remains low for women over 42, a statistic that has not improved in 25 years despite innovation [4–7].

DOR is a biologically heterogeneous syndrome that reflects the accelerated depletion of the ovarian follicular pool relative to age. The last decades have seen dramatic improvements in defining DOR, beginning with variable, often arbitrary clinic-based criteria [8, 9], and culminating in the current, more nuanced consensus frameworks (the Bologna and Poseidon criteria) [10, 11]. Nonetheless, even with standardization of entry criteria, most research cohorts remain highly variable with respect to both age and true risk. Critically, the overwhelming influence of age-related oocyte aneuploidy (euploidy/aneuploidy) on IVF prognosis has been under appreciated in research design and clinical counseling, perpetuating the myth that protocol can compensate for biology.

The etiology of DOR spans a spectrum: congenital syndromes, iatrogenic depletion, chronic inflammatory or autoimmune disorders, and, most commonly, “normal” reproductive aging [12].

Compounding these biological realities are multiple layers of clinical and methodological confusion: the misuse of surrogate endpoints (egg yield, blastulation rate) in place of live birth; the overinterpretation of small, underpowered studies; and the persistent belief, unsupported by data that “something must work”. In practice, even the best stimulation protocols cannot rescue the prognosis for women beyond a certain age or with substantially depleted ovarian reserve. Meanwhile, the emotional, psychological [13], and financial burdens carried by these patients are immense, and the proliferation of expensive, poorly supported “add-on” therapies presents a growing ethical challenge [14].

## Ovarian aging and decreased ovarian reserve

Human females are endowed at mid-gestation with approximately 6–7 million oocytes [15]. By the time of birth, only about 1–2 million remain, and this number falls further to roughly 300,000–400,000 at puberty [12]. From menarche onward, ovarian reserve, the pool of primordial follicles capable of generating a dominant follicle, declines unavoidably with age, progressing toward menopause (usually at 50–51 years), when the follicle count drops below the threshold required for regular ovulation.

A striking feature of human reproductive biology is that oocytes do not renew; the original pool is continuously depleted by a combination of ovulation and, far more significantly, atresia, a process of programmed cell death affecting both growing and dormant follicles throughout reproductive life [16]. While only some 400 oocytes are ultimately ovulated in a typical female lifetime, tens of thousands are lost yearly to atresia in the earlier reproductive years.

Ovarian reserve thus refers both to the quantity of follicles available and, indirectly, to the quality of remaining oocytes: the older the oocyte, the greater the probability of DNA damage, aneuploidy, and impaired developmental competence [16]. Advanced maternal age, then, is tightly linked to declining ovarian reserve, not only because fewer follicles remain, but also because those that do are intrinsically less competent to yield a healthy child.

### Measuring ovarian reserve: tools and pitfalls

For decades, the search for a reliable ovarian reserve test (ORT) has dominated reproductive endocrinology [17]. Three principal clinical tools remain in use: Antral Follicle Count (AFC); Anti-Müllerian Hormone (AMH) [18]; and Baseline FSH and Estradiol (E2). Despite these tools, the predictive value of any single ORT is limited when assessed independently of age. A low AMH at 27 and at 43 has vastly different prognostic import because oocyte quality remains higher in the younger woman [19, 20].

Of critical importance, AFC and AMH exhibit both inter- and intra-cycle variability: fluctuations of up to 30–60% have been described, especially in women with DOR [21]. This presents substantial challenges in reliably diagnosing DOR based on a single measurement or cycle. False labeling can result in unnecessary intervention or, conversely, unrecognized risk.

An additional important distinction should be made between DOR as a predictor of ovarian response during ART and its relationship with natural fertility. Although biomarkers, such as AMH, AFC, and basal FSH, reliably predict ovarian response to stimulation and the number of oocytes retrieved, their ability to predict spontaneous conception is considerably more limited. Several studies have demonstrated that, after adjustment for age, women with low AMH may achieve natural pregnancy rates comparable to those with normal ovarian reserve [22, 23], suggesting that ovarian reserve reflects the quantity of the remaining follicular pool rather than oocyte quality or reproductive competence. In contrast, in the setting of ART, reduced ovarian reserve is consistently associated with a poorer response to stimulation, fewer retrieved oocytes, and fewer embryos available for transfer or cryopreservation, which may ultimately decrease cumulative live birth rates despite preserved embryo quality, particularly in younger women. Therefore, DOR should not be considered synonymous with impaired natural fertility, but rather as a marker of reduced reproductive potential that is substantially more informative for predicting ovarian response to stimulation than spontaneous fecundity.

### Mechanisms of oocyte aging and aneuploidy

The qualitative decline of oocytes is as central as quantitative depletion in explaining the poor outcomes of ART in DOR and older women. As oocytes age, the proteins (cohesins) that hold sister chromatids together during meiosis degrade, increasing risks of non-disjunction and aneuploid embryos [24]. Oocytes arrested in prophase for decades accumulate DNA damage, and capacity for repair wanes with maternal age. Moreover, age-related mutation and damage of mitochondrial DNA impairs the high-energy requirements of meiosis, spindle formation, and early embryonic development [25].

This age-driven increase in aneuploidy underpins the “biological ceiling” for ART outcomes; DOR is largely a reflection of accelerated or advanced depletion of this competent oocyte pool [6, 26].

## Clinical definitions: from chaos to consensus

Until recently, the literature on DOR was a patchwork of in-house definitions, often varying not only between different centers, but sometimes within the same institution across time. Studies variously defined DOR using disparate cutoffs for AMH, AFC, day 3 FSH, or as "unexpectedly" poor oocyte yield with no requirement for repeatability [9]. Sometimes a single failed cycle sufficed for DOR labeling, while other protocols demanded multiple episodes. The age criterion was used chaotically: a 29-year-old and a 43-year-old might both be called "poor responders" in the same study. This heterogeneity in diagnostic approach rendered inter center and international comparisons nearly impossible and rendered meta-analyses deeply flawed. Most landmark studies on stimulation protocols and adjuvants in the 1990s and early 2000s recruited patients matching vastly variable "DOR" inclusion criteria, resulting in highly heterogeneous, underpowered, and mutually contradictory findings.

### The Bologna criteria (2011)

In an attempt to harmonize the field, ESHRE convened a consensus meeting, resulting in the "Bologna Criteria" [10]. According to Bologna, DOR (now formally designated as "poor ovarian response") was defined by the presence of at least two of the following: advanced maternal age (≥ 40 years) or any other risk factor for poor ovarian response; a previous poor ovarian response (≤ 3 oocytes with a conventional stimulation protocol); and an abnormal ovarian reserve test (AFC < 5–7 follicles or AMH < 0.5–1.1 ng/mL). In theory, this was expected to render research cohorts more uniform, allowing for valid trial comparison and meta-analysis.

Yet, some pitfalls remained: The inclusion of age was "optional", two women with entirely different reproductive prognoses could still both be labeled DOR; one episode of poor response sufficed for inclusion, despite known cycle-to-cycle biological variation [27]; AFC and AMH thresholds were not universally agreed upon; ROC curves differ by assay and population.

### The Poseidon criteria (2016)

In 2016, the Poseidon Group proposed further refinement [11]. They introduced subgroups based on age (below or above 35), ovarian reserve markers (AMH, AFC) and oocyte yield in prior cycles, with the goal of predicting the likelihood of obtaining at least one euploid embryo. While more biologically plausible, even the Poseidon criteria suffer from center-to-center variability and lack of universal application in research. Definitions of “abnormal” ORT are not fixed globally, and operationalization still varies.

### Clinical implementation and limitations

Despite their appeal, both Bologna and Poseidon have not been universally adopted in routine reporting, particularly outside academic centers or large prospective trials. Retrospective studies often apply their own DOR thresholds post hoc, and even recent RCTs intermix populations with different degrees of ovarian aging.

Moreover, these frameworks do not address underlying heterogeneity in etiology (e.g., iatrogenic, genetic, idiopathic), nor do they incorporate functional assessment (e.g., cumulative response over multiple cycles, egg maturity, fertilization rates), nor aspects of embryo competence.

### The role of age, oocyte quality, and embryo euploidy

Since more patients delay their family planning and rely on IVF to solve infertility, and while younger patients accomplish the mission quicker, the older ones with lower chances for live birth keep trying, the mean age of patients in IVF clinics keeps rising.

#### Age as the master variable in ART prognosis

It is now accepted beyond dispute that maternal age is the dominant predictor of reproductive success, not only in spontaneous conception, but especially in IVF/ICSI cycles for DOR. Age is strongly, independently, and non-linearly associated with cumulative live birth rate (cLBR), miscarriage risk, and the capacity to generate a chromosomally normal (euploid) embryo [6, 28].

This relationship is profoundly nonlinear. From puberty to about age 31–32, the monthly probability of conception is stable or even slightly rises [16]. After age 32, a slow decline begins, followed by an exponential drop from age 37 onward, just as the follicle pool accelerates its depletion [12, 16]. This “second slope” of decline correlates directly with the loss of high-quality (euploid) oocytes.

#### The dominance of embryo euploidy

The principal biological mechanism underlying age effects is embryo euploidy, the likelihood that an embryo carries a normal chromosome set. Large meta-analyses of PGT-A cycles show embryo euploidy rates among blastocysts are roughly > 60% for women < 35; 40% for women 38–40 and 25% for women 41–42.

[28]. Irani et al. [6] studied a total of 12,298 embryos for the ploidy status. Women were divided into five age groups < 35, 35–37, 38–40, 41–42, and > 42 years old. The euploidy rate dropped from 55.4%, 44.8%, 32.4%, 18.2% to 8.5%, respectively. Notably, euploidy rates were unaffected by total gonadotropin dosage, stimulation duration, number of oocytes harvested, size of the largest follicles, or peak estradiol levels [6].

Almost all women under 36 who achieve a euploid embryo and undergo single embryo transfer eventually achieve live birth, LBR per transfer of a euploid embryo > 60%; cumulative LBR after 2–3 euploid embryos is over 90% [29]. In contrast, the number of mature oocytes required to obtain at least one euploid blastocyst increases substantially with advancing maternal age [30], women aged > 42 may need > 30 mature oocytes for an 80% probability of achieving at least one euploid embryo.

The impact of DOR is multiplicative with age. At a young age, a woman with DOR may simply require more cycles or retrieve fewer eggs per cycle but, as long as a euploid embryo is generated, her true prognosis is robust [5]. At advanced age, even a woman with “normal” reserve faces poor odds, and those with DOR have a very small probability of producing a transferable, chromosomally normal embryo [6].

To conclude, all current high-quality studies, including data from PGT-A, clinical cohort studies, and cumulative outcome analysis, agree that maternal age, not DOR per se, not protocol, nor adjuvant, determines the major probability of live birth. Grouping or analyzing DOR without stringent age stratification is a flawed research design and a disservice to patients.

### Young women with diminished ovarian reserve

Young women with DOR represent a distinct subgroup whose reproductive outcomes differ substantially from those of older women with similar ovarian reserve parameters. Although low AMH, reduced AF), or elevated basal FSH predict a reduced ovarian response to stimulation and fewer oocytes retrieved, these biomarkers do not reliably reflect oocyte quality. As maternal age remains the primary determinant of oocyte competence and embryonic aneuploidy, younger women with DOR generally have higher implantation, clinical pregnancy, and live birth rates per embryo transfer than older women with comparable ovarian reserve. Nevertheless, the lower oocyte yield may result in fewer embryos available for transfer or cryopreservation, potentially reducing cumulative live birth rates and increasing the need for multiple stimulation cycles. Therefore, counseling should emphasize that DOR in young women primarily reflects diminished oocyte quantity rather than compromised oocyte quality, while also highlighting the potential for a shortened reproductive lifespan due to accelerated ovarian depletion.

Management of young women with DOR should focus on timely reproductive planning and individualized treatment strategies aimed at maximizing the number of competent oocytes retrieved. Controlled ovarian stimulation remains the standard approach, although no stimulation protocol has consistently demonstrated superiority in this population. For selected patients, repeated stimulation cycles or oocyte/embryo accumulation may improve cumulative reproductive outcomes, whereas double stimulation (DuoStim) may be considered when rapid oocyte collection is desirable, although evidence supporting improved live birth rates remains limited [31]. Adjunctive treatments, including dehydroepiandrosterone (DHEA), transdermal testosterone, and growth hormone, have shown inconsistent results and are not currently recommended for routine use because high-quality evidence demonstrating improved live birth rates is lacking [2]. Consequently, early referral, individualized counseling, and realistic expectations remain central to the management of young women with DOR, while further age-stratified studies are needed to define the most effective therapeutic strategies.

### The limits of ovarian stimulation: “more eggs ≠ more babies” at older ages

The longstanding dogma, “more eggs, more chance”, is true only up to a moderate oocyte yield and younger ages [1, 32]. It is generally accepted that the success rate in IVF is directly related to the number of eggs collected, at least up to 15–20 eggs [1, 33], after which it plateaus or might even decrease. A retrospective study found that in DOR patients, the increased number of oocytes retrieved from 1–3 to > 3, is the only independent variable significantly associated with increased LBR [34]. Also, a large pivotal study (*N* = 400,135 cycles) supports the finding that increasing the oocyte yield from 2 to 3 resulted in a 25% relative increase in LBR across all age groups [1].

These retrospective associations do not imply that we can actively increase egg numbers by simply manipulating the FSH dose. Clinicians often individualize the FSH dose based on women’s age and ovarian reserve tests; however, it has not been proven that this approach improves clinical outcomes [35]. The change in egg number from cycle to cycle might be due to random fluctuations unrelated to the administered dose.

Protocols for stimulating IVF in normal responders are straightforward. Although no proven superiority has been shown between doses, a daily dose of 100–250 IU is common. The ovarian stimulation protocols employed for DOR are more complex, using higher doses of gonadotropins ranging up to 600 IU and more [36, 37]. The only clear conclusion [38] is that none of the many protocols tested with various FSH doses proved superior to standard stimulation. Moreover, a large retrospective study (*N* = 658,519) reported that daily FSH or rFSH dosing > 300 IU significantly decreased the odds of a live birth [32]. Van Tilborg et al. [39] reported that increasing the FSH dose from 150 to 225 or 450 IU/day in women with a predicted lower reserve (AFC < 8 compared to > 8–10) does not improve cumulative LBR in IVF, suggesting that a lower dose than customarily used for DOR may be appropriate. An AFC of less than 8 is considered a medium response and, therefore, cannot be trusted to lead the discussion on reducing the dose to treat DOR to 150 IU. Whether the dose should be adjusted after the initial response or kept constant throughout stimulation is another source of disagreement [40].

#### Cycle-to-cycle variability and randomness

Ovarian recruitment is not only a function of subject or protocol, but also of stochastic follicle wave dynamics [21, 41]. Two consecutive cycles in the same woman may yield divergent egg numbers and embryo counts; in DOR, with only a handful (or fewer) primordial follicles in reserve, random “good” or “bad” cycles may have enormous effect. Klinkert et al. [27] showed that among so-called unexpected poor responders, 45 of 120 patients (38%) continued IVF treatment after the first cycle became pregnant in the second or third cycle; most of them had a normal response.

The normal biological variability in AFC and AMH is 30–60%, so one-off “failures” may reflect chance [21]. This again argues for a requirement of two or more consecutively poor responses before DOR is diagnosed, especially at younger ages.

#### The myth of stimulation protocols and add-ons

Ever since the first IVF cycles, reproductive medicine embraced the assumption that “more is better”: if low oocyte yield limits cumulative live birth, then increasing stimulation intensity, especially FSH doses, must remedy poor outcomes. This philosophy fostered the habitual use of ever higher gonadotropin doses in DOR patients, sometimes exceeding 450–600 IU per day [37], the introduction of GnRH-agonist “flare” protocols, microdose regimens, and an array of experimental adjuvants [2, 42].

No head-to-head protocol, antagonist, long agonist, microdose flare, “Duo-Stim”, “short” or “stop” protocols, has demonstrated reproducible, statistically robust superiority in LBR for DOR patients [38]. Pretreatment medications/manipulations are offered to patients with poor ovarian response, aiming to prevent the occurrence of a spontaneous luteinizing hormone surge, increase the number of preantral and antral follicles, synchronize follicular development, and increase oocytes’ yield and quality, with the consequent improvement in cycle outcome. Although early small human studies were encouraging, interpretation of the studies is compromised by variations in the type, dose, combination and duration of the pretreatment medications [2]. Moreover, many protocols and add-ons are justified based on process parameters, egg or embryo count, fertilization or blastulation rates, endometrial thickness, etc. rather than cumulative live birth. Improvements in laboratory endpoints do not reliably translate into patient-important outcomes if embryo competence does not increase.

Despite the early optimism and a plethora of positive case series, the expected breakthrough has not materialized. Disillusionment has grown as larger, methodologically robust investigations consistently fail to demonstrate meaningful improvements in live birth rates.

### Methodological pitfalls and wasted efforts

Underpowered studies, multiple comparisons and selective reporting, bias toward publishing positive findings, and industry enthusiasm all contribute. Many “successes” in small cohorts fail to replicate in large samples or multicenter trials. The field is rife with treatments that “make sense” physiologically, but do not change outcomes when properly studied.

#### The reproducibility crisis in reproductive medicine

Infertility research, and DOR studies in particular, have been beset by what ESHRE’s 2018 Capri Workshop Group lamented as a scandalous wastage of research resources: "85% of all research funding is wasted due to inappropriate research questions, faulty study design, flawed execution, irrelevant endpoints, poor reporting, or non-publication" [43, 44]. DOR research is emblematic of each of these problems.

#### Cohort heterogeneity: the age confounder

Age as a Prognostic "Super-Variable": Perhaps the single most pervasive error in DOR research is the failure to properly stratify or control for age within study cohorts. Most trials and nearly all retrospective series, especially before 2017, group all DOR women together, regardless of whether they are 29, 37, or 44 yrs old. This practice dramatically dilutes statistical power. Age is so dominant that any "protocol effect" is dwarfed by inherent biological differences. The result? True effects are masked ("Type II errors"), and false associations (e.g., improved lab endpoint) are mistaken for clinically meaningful results.

#### The surrogate endpoint trap

Research routinely prioritizes surrogate/laboratory endpoints over what matters to patients, e.g. number of follicles, eggs, blastocysts; fertilization or blastulation rates; implantation rate (often per embryo, not per cycle or patient). While easy to collect and analyze, these data do not always translate into live birth, particularly when age and embryo competence are not accounted for.

#### Lack of repeated poor response criterion

An additional flaw in many DOR studies is the acceptance of single-cycle "poor response" as an entry criterion. Given high natural variability, approximately 35–40% of such women will experience normal response in a subsequent cycle [27] Therefore, ≥ 2 consecutive poor response cycles, under well-defined protocols, should be an entry requirement, particularly for interventional clinical trials.

#### Power, sample size, and multicenter need

DOR with strict, repeatable criteria and tight age bands is uncommon; many studies are woefully underpowered. Most single-center studies lack sufficient numbers per arm to detect any, but the largest effects. Negative studies are thus often Type II false negatives; positive, small studies typically fail to replicate. Harmonized multicenter collaborations, shared protocols, and common data elements are, therefore, required to obtain reliable, generalizable insights.

#### Publication bias and selective reporting

Numerous "breakthrough" add-ons in DOR (e.g. growth hormone, DHEA, scratching, mitochondrial supplements) first appeared in small positive series, but large, properly powered studies or registry data failed to confirm LBR benefit [45, 46]. Moreover, while positive trials are more likely to be published, especially in "hot" topics or where commercial interests predominate, negative or null results remain "file-drawered", biasing meta-analyses and fostering illusory optimism about interventions or protocols.

The last decade has seen an explosion of meta-analyses in reproductive medicine, more, sometimes, than randomized controlled trials themselves. Yet, unless constituent studies are well coded for age, DOR definition, and live birth as outcome, meta-analyses merely amplify heterogeneity and confusion [47]. For example, multiple "meta-analyses" on DHEA or GH adjuvant therapy for DOR reach contradictory conclusions, as different inclusion/exclusion rules, outcome definitions, or endpoints are used.

## Conclusions

After four decades of clinical research, innovation, technical refinement, and enormous patient investment, in both time and resources, the field of assisted reproduction stands at a sobering crossroads regarding DOR. Despite technological advancement and the proliferation of both stimulation protocols and laboratory “add-ons,” live birth rates in women diagnosed with DOR have remained stubbornly static, especially among patients of advanced maternal age. The persistent hope, shared by patients, clinicians, and the ART innovation industry alike, that biology can be readily overcome by altered protocols, new adjuvants, or ever-increasing hormonal dosages, has not materialized in improved real-world outcomes [36]. The major limiting factor to live birth rate in DOR is not gonadotropin dose, stimulation protocol, or the sophistication of the laboratory. It is the sharp, age-dependent exponential decrease in the probability of obtaining at least one euploid embryo. Oocyte, and, therefore, embryo, competence is dictated by the biology of the ovary and is primarily a function of age, not egg number, not laboratory method, and not incremental hormonal adjustment.

It is suggested that future studies should (1) stratify cohorts into narrow, 1–2-year age brackets to more accurately assess age-related biological effects; (2) require at least two episodes of poor ovarian response for DOR diagnosis, minimizing the confounding impact of natural cycle-to-cycle variability; (3) move beyond surrogate outcomes and prioritize live birth as the primary endpoint; and (4) focus on large, multicenter trials to enable statistically significant data collection for the relatively uncommon DOR population within narrowly defined age ranges.

If these methodological improvements are realized, future research may help identify meaningful advances while providing patients with realistic counseling about their prognosis and the diminishing returns of current interventions with advancing age.

## Data Availability

No datasets were generated or analysed during the current study.
